# Autophagy‐Sirt3 axis decelerates hematopoietic aging

**DOI:** 10.1111/acel.13232

**Published:** 2020-09-20

**Authors:** Yixuan Fang, Ni An, Lingjiang Zhu, Yue Gu, Jiawei Qian, Gaoyue Jiang, Ruijin Zhao, Wen Wei, Li Xu, Gaochuan Zhang, Xingyun Yao, Na Yuan, Suping Zhang, Yun Zhao, Jianrong Wang

**Affiliations:** ^1^ Hematology Center of Cyrus Tang Medical Institute Jiangsu Institute of Hematology Institute of Blood and Marrow Transplantation Collaborative Innovation Center of Hematology National Clinical Research Center for Hematologic Diseases, The First Affiliated Hospital Soochow University School of Medicine Suzhou China; ^2^ Department of Hematopoietic Engineering Susky Life SciTech (Suzhou) Co., LTD. Suzhou China; ^3^ State Key Laboratory of Radiation Medicine and Radioprotection Soochow University School of Medicine Suzhou China; ^4^ School of Biology and Basic Medical Sciences Soochow University Suzhou China

## Abstract

Autophagy suppresses mitochondrial metabolism to preserve hematopoietic stem cells (HSCs) in mice. However, the mechanism by which autophagy regulates hematopoietic aging, in particular in humans, has largely been unexplored. Here, we demonstrate that reduction of autophagy in both hematopoietic cells and their stem cells is associated with aged hematopoiesis in human population. Mechanistically, autophagy delays hematopoietic aging by activating the downstream expression of Sirt3, a key mitochondrial protein capable of rejuvenating blood. Sirt3 is the most abundant Sirtuin family member in HSC‐enriched population, though it declines as the capacity for autophagy deteriorates with aging. Activation of autophagy upregulates Sirt3 in wild‐type mice, whereas in autophagy‐defective mice, Sirt3 expression is crippled in the entire hematopoietic hierarchy, but forced expression of Sirt3 in HSC‐enriched cells reduces oxidative stress and prevents accelerated hematopoietic aging from autophagy defect. Importantly, the upregulation of Sirt3 by manipulation of autophagy is validated in human HSC‐enriched cells. Thus, our results identify an autophagy‐Sirt3 axis in regulating hematopoietic aging and suggest a possible interventional solution to human blood rejuvenation via activation of the axis.

## Graphical abstract

Autophagy collaborates with Sirt3, to form a regulatory axis that synergistically delays hematopoietic aging in both mice and humans. Positive intervention to the autophagy‐Sirt3 axis leads to blood rejuvenation.

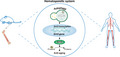

## 1 INTRODUCTION

Aging in hematopoiesis is strongly associated with acquired hematopoietic malignancies such as bone marrow myelodysplastic syndrome, myeloproliferative disorders, and leukemia (Adams, Jasper, & Rudolph, 2015; Chung & Park, 2017; Genovese et al., 2014; Jaiswal et al., 2014; Steensma et al., 2015). Hematopoietic aging features a limited potential for reconstitution of hematopoietic stem cells (HSCs), reduced production of red blood cells and lymphocytes, as well as myeloid‐biased differentiation (Chung & Park, 2017; Elias, Bryder, & Park, 2017; Latchney & Calvi, 2017; Morrison, Wandycz, Akashi, Globerson, & Weissman, 1996; Pang et al., 2011). Hematopoietic aging is often associated with a clonal shift in the HSC pool and subsequent clonal hematopoiesis caused by cell‐intrinsic and cell‐extrinsic factors (Chung & Park, 2017). Recent study shows that in mice, inflammation promotes HSC aging via IL27Ra (He et al., 2020). However, the cellular and molecular basis responsible for hematopoietic aging, in particular in humans, remains largely unclear.

Autophagy is a highly conserved metabolic process that is critically essential to maintain cellular homeostasis under normal and stress conditions, as its activation promotes cellular survival by limiting oxidative stress, maintaining adequate metabolic functions, and controlling bioenergetic levels and amino acid pools. In mammalian species, autophagic machinery is controlled by several autophagy‐essential protein complexes, such as the microtubule‐associated protein light chain 3 (LC3) conjugation system. It includes LC3 proteins and γ‐aminobutyric acid receptor‐associated proteins (GABARAPs), and the ATG12 conjugation system that includes the ATG12‐ATG5‐ATG16L1 complex. ATG7 is a critical component in maintaining the function of the above two conjugation systems, and loss of ATG7 disables the formation of double‐membrane autophagosomes, thus blocking the initiation of autophagy (Mizushima, Levine, Cuervo, & Klionsky, 2008). Autophagy is required for the maintenance of HSC pool (Liu et al., 2010; Mortensen et al., 2011), HSC mobilization (Leveque‐El Mouttie et al., 2015), and multilineage differentiation (Cao, Cai, et al., 2015; Mortensen et al., 2011)), and it also secures hierarchical organization of the hematopoietic system (Cao, Zhang, et al., 2015).

Since aging results from the progressive accumulation of cellular damage by chronic insults, autophagy, as a sensor of multiple stresses, has been linked to aging. Previous studies using loss‐of‐function mutations in autophagy genes in animal models such as yeast, *C*.* elegans*, flies, and mice have shown that alteration to autophagy reduces their life span, whereas activation of autophagy prolongs life span in yeast and mice (Fernandez et al., 2018; Hansen, Rubinsztein, & Walker, 2018; Rubinsztein, Marino, & Kroemer, 2011). A recent study in mouse models shows that autophagy is critical for protecting HSCs from metabolic stress by clearing active, healthy mitochondria to maintain their quiescence and potency through the mitophagy pathway (Ho et al., 2017). Loss of autophagy in HSCs causes accumulation of mitochondria and a constitutively activated metabolic state, which drives accelerated myeloid differentiation and impairs the capacity of HSCs for self‐renewal (Ho et al., 2017).

Sirtuins are nicotinamide adenine dinucleotide (NAD^+^)‐dependent protein deacetylases (Katsyuba, Romani, Hofer, & Auwerx, 2020). Several members of the Sirtuin family have been shown to regulate aging or life span in numerous lower organisms including yeast, nematodes, and fruit flies (Burnett et al., 2011; Haigis & Guarente, 2006), as well as higher organisms such as mice (Kanfi et al., 2012). In the process of aging, Sirtuins have been reported to be related to, but not limited to their roles in regulation of energy metabolism, response to calorie restriction, and control of cell death (Chang & Guarente, 2014; Guarente, 2013). Most of the Sirtuin family members have been documented in the regulation of mammalian hematopoietic function, in which Sirt2, Sirt3, and Sirt7 were found reduced transcription in aging HSCs (Chambers & Goodell, 2007), Sirt1 controls HSC homeostasis via the longevity transcription factor FOXO3 (Rimmele et al., 2014), Sirt6 controls HSC homeostasis through epigenetic regulation of Wnt signaling (Wang et al., 2016)), and Sirt7 is implicated in HSC aging via mitochondrial unfolded protein response that ensures proteostasis in the mitochondria (Mohrin et al., 2015; Mohrin, Widjaja, Liu, Luo, & Chen, 2018). In particular, Sirt3 reverses aging‐associated HSC degeneration by regulating the global acetylation landscape of mitochondrial proteins and effectively reducing oxidative stress (Brown et al., 2013). Sirt3 is indispensable in aged mice, and its expression is inhibited in aging (Brown et al., 2013). However, the regulatory means by which Sirt3 is suppressed during aging remains unknown.

Although autophagy and Sirt3 have been implicated in hematopoietic aging, the mechanisms underlying their roles have just been begun to explore in model mammals. In particular, the functional interconnection between autophagy and Sirt3 in human hematopoietic aging and the solution to rejuvenating human blood remain fundamentally unexplored. In this study, we show that autophagy cooperates with Sirt3 by promoting its expression to decelerate hematopoietic aging and that positive intervention to autophagy‐Sirt3 axis leads to blood rejuvenation.

## 2 RESULTS

### 2.1 Hematopoietic autophagy activity is reduced in aged human population

**FIGURE 1:**
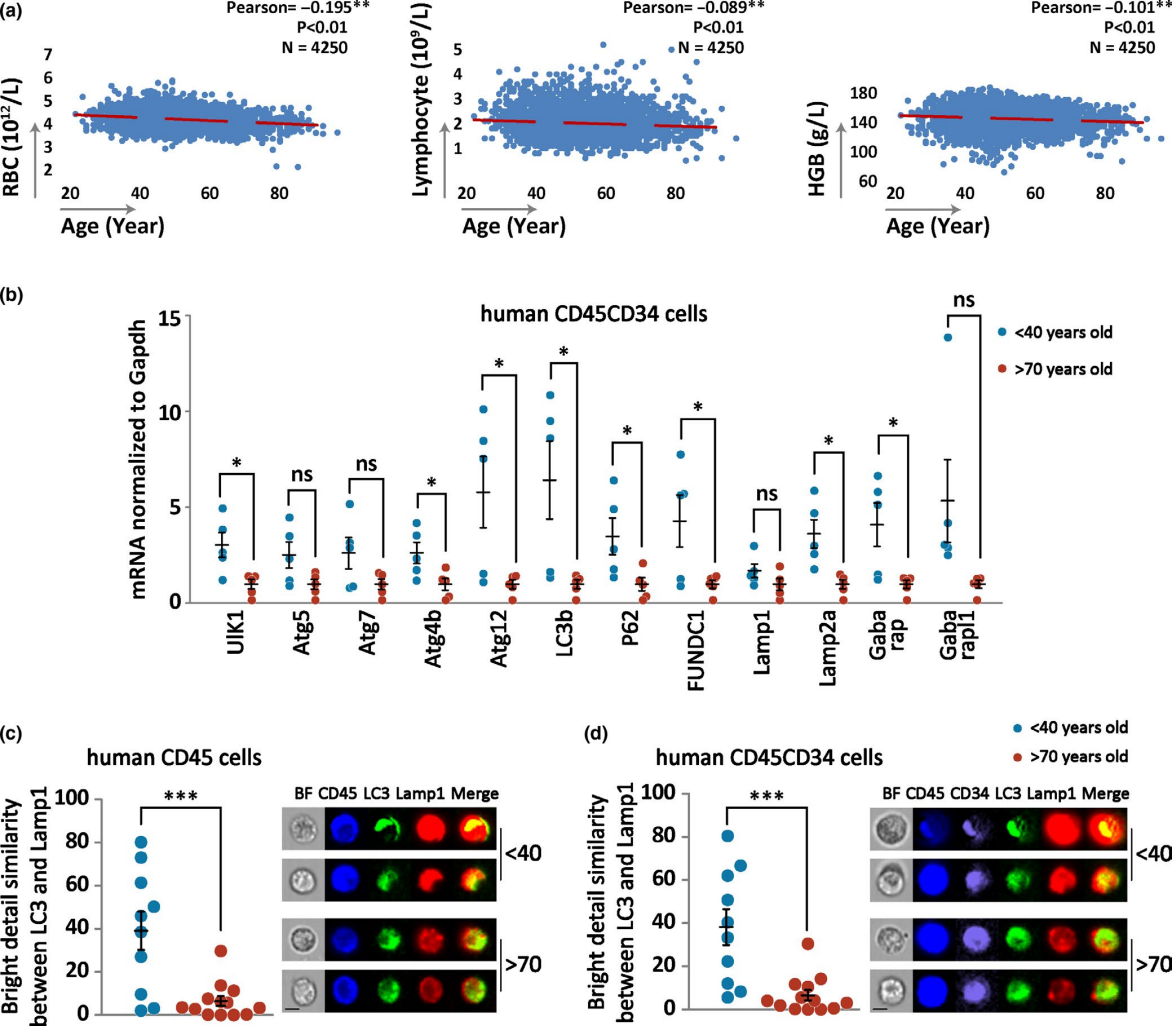
Reduction of autophagy is associated with aged hematopoiesis in human population. (a) Linear regression and Pearson correlation coefficients of peripheral blood counts in aging human population. A pool of peripheral blood count information from physical examination of 4250 people aged from 20 to 90 years was analyzed using SPSS statistic software. (b) Quantitative PCR measuring the expression of autophagy‐essential genes in human bone marrow primary HSC‐enriched hematopoietic cells (CD45CD34) from the indicated two age groups. (c, d) Quantitative ImageStream detection of basal autophagy levels in human bone marrow primary hematopoietic cells (CD45), HSC‐enriched hematopoietic cells (CD45CD34) from the two age groups. Left, statistical data from individual human samples. Right, representative images of the cells, either single‐stained (CD45—blue; CD34—purple; LC3—green; Lamp1—red) or stained for both markers (merge of LC3 and Lamp1). Bar, 10 μm

Human peripheral blood analysis of a population of 4250 individuals ranging in age from 20 to over 90 years showed a progressive decrease in hemoglobin levels, as well as red blood cell (RBC) and lymphocyte counts with increase of age (Figure 1a), which are hallmarks for hematopoietic aging (Chung & Park, 2017; Latchney & Calvi, 2017; Pang et al., 2011). Recent studies in several model organisms demonstrate that autophagy declines with aging (Burnett et al., 2011; Chung & Park, 2017). While the age‐related decline in autophagy has been documented in mammalian animal cells, direct association between autophagy and hematopoietic aging in humans has not been studied. Fluorescently tagged or endogenous LC3/GABARAP family proteins are commonly used markers for autophagy in higher eukaryotes to facilitate microscopic visualization of phagophores, autophagosomes, or autolysosomes in the cell. Use of a second marker, and depending on what type of the marker, can reveal initiation of autophagy or functional autophagy when autophagic targets are degraded in the autolysosomes (Mizushima et al., 2008). To search for direct evidence linking autophagy to blood aging in human subjects who were not in medical intervention, we examined the physiological activity of autophagy in primary human blood cells from two age groups. Expression of genes encoding autophagy machinery was highly reduced in the HSC‐enriched hematopoietic cells in the aged group (over 70 years old) compared with the young group (below 40 years old) (Figure 1b). To further address the impact of functional autophagy on human blood aging, we applied image flow cytometry to observe co‐localization of LC3 and the lysosomal marker Lamp1 (Phadwal et al., 2012) for measuring activity of hematopoietic autophagy during autolysosome formation in the two differently aged populations. The results show that in comparison with the young group, there was a significant decrease in basal autophagy levels in both total bone marrow hematopoietic cells (CD45‐positive) and HSC‐enriched hematopoietic cells (CD45CD34‐positive) in the aged human population (Figure 1c,d). Therefore, autophagy is significantly reduced in hematopoietic cells and their stem cells in the aged population of humans.

### 2.2 Loss of Atg7 in the hematopoietic system causes accelerated blood aging

**FIGURE 2:**
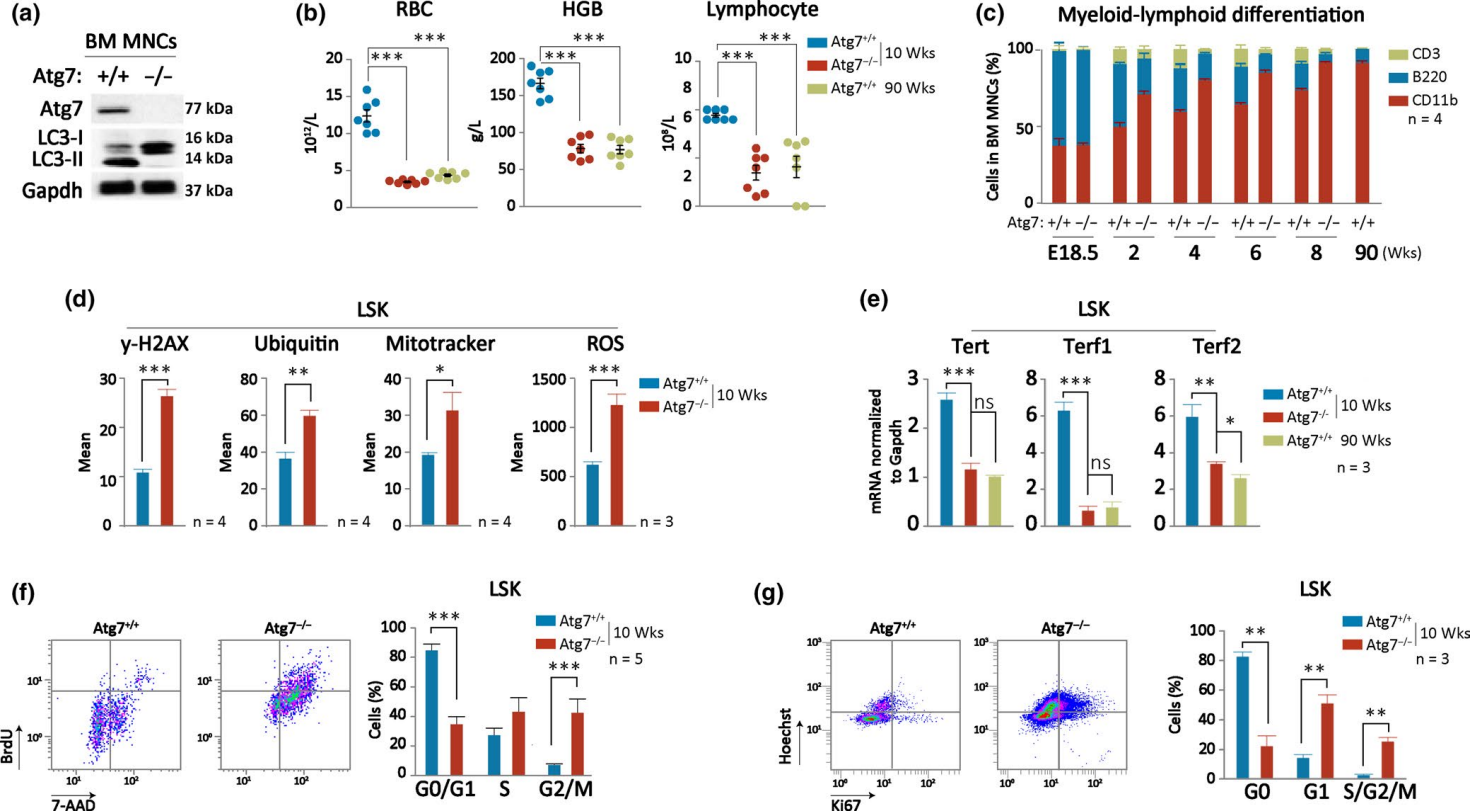
Deletion of autophagy‐essential gene Atg7 accelerates hematopoietic aging in mice. (a) Western blotting confirmation of autophagy disruption from Atg7 deletion in BM mononuclear cells of the Atg7^f/f^;Vav‐iCre mouse model. (b) Comparison of peripheral blood counts between autophagy‐defective mice (Atg7^−/−^) and wild‐type (young or aged) mice (Atg7^+/+^). (c) Flow cytometric analysis of myeloid‐biased hematopoietic differentiation in autophagy‐defective mice and wild‐type mice (young or aged). (d) Flow cytometric detection of metabolic stress levels in the HSC‐enriched cells of wild‐type mice (young or aged) and Atg7‐deleted mice. Total cellular ROS was measured. (e) Quantitative PCR measurement of telomerase expression in the HSPCs from autophagy‐defective mice and wild‐type mice (young or aged). Primer information is listed in Table S2. (f, g) Flow cytometric analysis on the cell cycle of the HSC‐enriched cells from wild‐type and Atg7‐deleted mice. Left, representative flow density plot. Right, statistic data showing distribution of the percentage of HSC‐enriched population in their cell cycle

To study the role of autophagy in hematopoietic aging, we used an Atg7^f/f^;Vav‐iCre mouse model in which the Atg7, an essential autophagy gene, is deleted (Atg7^−/−^) primarily in hematopoietic cells and their stem and progenitor cells (Figure 2a). Deletion of Atg7 blocks the formation of both ATG8 and ATG12 systems, completely disabling canonical autophagy (Cao, Zhang, et al., 2015; Mizushima et al., 2008; Nishida et al., 2009). At 10 weeks old, mice carrying the Atg7 deletion displayed a significant decrease in their RBC and lymphocyte counts, as well as in their level of HGB, comparable to that of 90‐week‐old mice (Figure 2b). Atg7 deletion in the mouse model also accelerated biased myeloid–lymphoid differentiation (Figure 2c), which is the most recognized hallmark for hematopoietic aging (Chung & Park, 2017; Elias et al., 2017; Latchney & Calvi, 2017; Pang et al., 2011). Protein homeostasis, mitochondrial accumulation, and generation of ROS and DNA damage are also hallmarks of cellular aging (Martin‐Pardillos et al., 2017; Pilzecker et al., 2017). Examination by flow cytometry showed that all of these parameters were increased in the Atg7‐deleted mouse HSC‐enriched population (Lin^−^c‐Kit^+^Sca‐1^+^, LSK) (Figure 2d). Telomere length is another important indicator for cell aging (Calado & Dumitriu, 2013). Telomere length is largely maintained by telomerase reverse transcriptases that catalyze the addition of bases to the end of the telomere (Lansdorp, 2017). We measured the expression of three telomerase genes, namely Tert, Terf1, and Terf2 in the HSC‐enriched cells by quantitative reverse transcription‐PCR, and found that expression of these telomerase genes was reduced in the hematopoietic Atg7‐deleted 10‐week‐old mice, comparable to their expression in the wild‐type mice at 90 weeks old (Figure 2e). Cell proliferation assays measured by flow cytometry displayed a decreased frequency of G0/G1 and an increased frequency of G2/M in the Atg7‐deleted HSC‐enriched cells (Figure 2f). Further analysis of cell cycle shows that in Atg7‐deleted HSC‐enriched population, cell frequency was decreased solely for cells in the G0 phase but increased in cells at the G1 and G2/M phases (Figure 2g), suggesting that Atg7 deletion leads to a lower percentage of HSC‐enriched cells in quiescence, which can eventually cause stem cell exhaustion and accelerated aging (Garcia‐Prat et al., 2016). Taken together, these results demonstrate that Atg7 deletion causes greatly accelerated hematopoietic aging, particularly in HSC‐enriched population.

### 2.3 Loss of Atg7 selectively suppresses Sirt3 in mouse hematopoietic system

**FIGURE 3:**
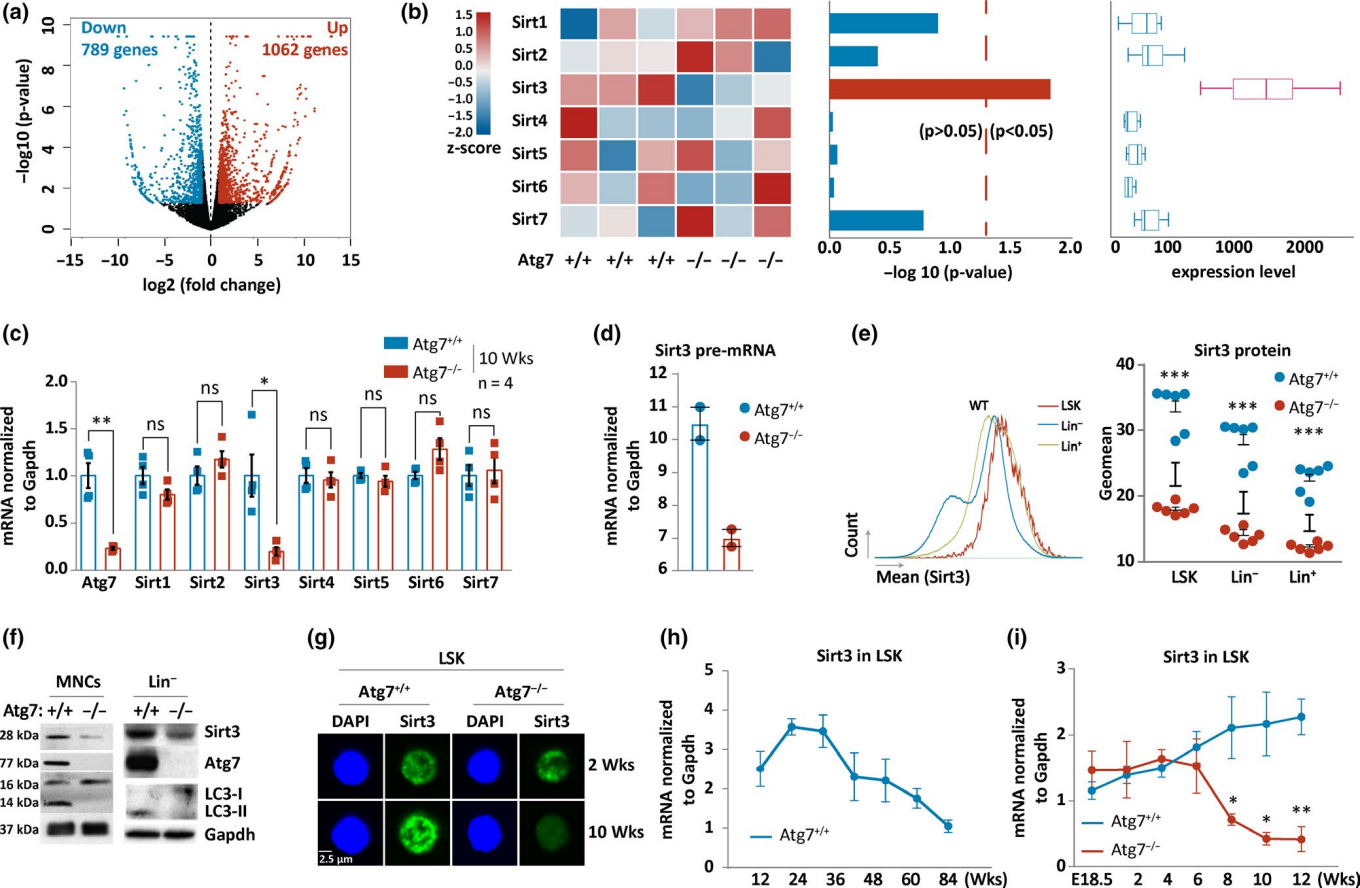
Sirt3 expression depends on Atg7 in the mouse bone marrow HSC‐enriched hematopoietic cells. (a) The volcano map of differentially expressed genes in Atg7^−/−^ HSC‐enriched hematopoietic cells as compared to Atg7^+/+^ HSC‐enriched cells. A total of 1062 genes were significantly upregulated, while 789 genes were downregulated in Atg7‐depleted HSC‐enriched cells. (b) RNA sequencing reveals Sirt3 dependency on Atg7. Left, the heatmap of Sirtuin family between Atg7^+/+^ and Atg7^−/−^ HSC‐enriched cells. The result shows that only Sirt3 expression is reduced in the HSPCs of all of the three Atg7^−/−^ mice. Middle, the reduction levels of the expression of seven Sirtuin family members in the HSC‐enriched cells due to Atg7 deletion. The result shows that only Sirt3 expression is significantly reduced in the Atg7^−/−^ HSC‐enriched cells. Right, the relative expression levels of Sirtuin family members in wild‐type HSC‐enriched cells. The result shows that Sirt3 is the highest expressed member in Sirtuin family in the HSC‐enriched cells of the wild‐type mice. (c) Measurement of expression levels of the Sirtuin family members by quantitative PCR in the Atg7^+/+^ and Atg7^−/−^ HSC‐enriched cells. (d) Measurement of Sirt3 pre‐mRNA by quantitative PCR. pre‐mRNA expression was normalized to gapdh level. (e) Flow cytometric examination of Sirt3 protein expression in HSC‐enriched cells, hematopoietic progenitor cells, and terminally differentiated hematopoietic cells of wild‐type and Atg7‐deleted mice aged 10 weeks. Left, representative data of Sirt3 protein expression levels in HSC‐enriched cells (LSK), HPCs (Lin^−^), and terminally differentiated hematopoietic cells (Lin^+^) by flow cytometry. Right, statistic data for comparison of Sirt3 expression levels. (f) Measurement of expression of Sirt3, Atg7, and LC3 proteins by Western blotting in Atg7^+/+^ and Atg7^−/−^ bone marrow mononuclear cells (MNCs) and hematopoietic progenitor cells (Lin^−^ sorted) from 10‐week‐old mice. Gapdh serves as a loading control. (g) Confocal detection of Sirt3 protein in the Atg7^+/+^ and Atg7^−/−^ HSC‐enriched cells. Representative images were taken from bone marrow HSC‐enriched cells of mice aged at 2 weeks and 10 weeks. (h) Quantitative PCR measurement of Sirt3 expression levels in the HSC‐enriched cells from aging wild‐type mice. (i) Time course comparison on Sirt3 expression by quantitative PCR in HSC‐enriched cells of wild‐type and Atg7‐deleted mice

To explore the mechanistic connection between Atg7 and hematopoietic aging, we performed RNA sequencing of HSC‐enriched population from wild‐type and Atg7‐deleted mice. The volcano map of differentially expressed genes between Atg7^+/+^ and Atg7^−/−^ HSC‐enriched cells of the mice revealed a total of 1062 significantly upregulated genes, with 789 downregulated genes in the Atg7‐depleted HSC‐enriched cells (Figure 3a). Sirtuins have long been recognized as important regulators of aging (Burnett et al., 2011; Haigis & Guarente, 2006; Kanfi et al., 2012). The transcriptomic profiling reveals that Sirt3 is the most transcriptionally abundant member in Sirtuin family (Figure 3b, left and middle panels), and deletion of Atg7 solely led to significant loss of Sirt3 expression, among all seven of the Sirtuin family genes (Figure 3b, right panel). Validation by quantitative PCR shows that Sirt3 expression is dependent on the presence of a functional Atg7, and hence possibly an intact autophagy system, whereas deletion of Atg7 does not alter the expression levels of the rest Sirtuin family members (Figure 3c). Further detection of pre‐mRNA by quantitative PCR discloses significant reduction in Sirt3 level in Atg7‐deleted HSPCs (Figure 3d), suggesting that the transcription of Sirt3 depends on Atg7 and the reduction in Sirt3 mRNA level due to Atg7 deletion is not caused by its mRNA instability. Notably, Sirt3 protein levels measured by flow cytometry are higher in the upper stages of the hematopoietic hierarchy since there is a progressive decrease in expression in the three hematopoietic populations that include HSC‐enriched cells (LSK), HPCs (hematopoietic progenitors, Lin^−^), and differentiated functional blood cells (Lin^+^), and Sirt3 dependency on Atg7 appeared to exist in the entire hematopoietic hierarchy (Figure 3e). Examination with Western blot analysis also shows reduced Sirt3 protein levels in bone marrow mononuclear cells (MNCs) and hematopoietic progenitor cells (Lin^−^) of the hematopoietic Atg7‐deleted mice (Figure 3f). Similarly, confocal microscopy reveals that in the Atg7‐deleted HSC‐enriched cells, Sirt3 protein maintains a relatively high level in 2‐week‐old mice, but by 10 weeks of age the Sirt3 levels are rapidly reduced (Figure 3g). The loss of Atg7 thus causes a significant decrease in Sirt3 expression by 10 weeks of age. In accordance with the reduction of Sirt3, RNA sequencing of the HSC‐enriched hematopoietic cells from 10‐week‐old mice discloses a decreased expression of an array of anti‐aging genes due to Atg7 deletion (Figure S1).

To further determine the expression pattern of Sirt3 in hematopoietic aging, we tracked a time course of Sirt3 transcription in wild‐type mice by quantitative RT‐PCR. The data show that Sirt3 increased its expression until 24 weeks and thereafter transcription progressively decreased in the HSC‐enriched cells (Figure 3h), a dynamic pattern similar to deterioration of autophagy in physiological aging. Surprisingly, Sirt3 expression began to dramatically decrease at 4–6 weeks in the mouse model in which hematopoietic Atg7 is disrupted (Figure 3i), a pattern almost identical to the accelerated hematopoietic aging due to the low activity of autophagy (Figure 1). In 10‐week‐old Atg7‐deleted mice, Sirt3 expression is lower than that of the wild‐type mice at 84 weeks (Figure 3h,i). Together, the above data demonstrate that Sirt3 expression depends on Atg7.

### 2.4 Loss of Atg5 causes loss of Sirt3 and aging of hematopoiesis, resembling Atg7 deletion

To answer whether Atg7 deletion‐caused reduction in Sirt3 expression and hematopoietic aging is autophagy‐dependent, we generated hematopoietic Atg5‐deleted mice by crossing the Atg5^flox^ mice from Dr. Noboru Mizushima, Japan (Hara et al., 2006), to Vav‐iCre mice (Jackson Laboratory) to examine the effect of deletion of Atg5, another autophagy‐essential gene, on Sirt3 in the hematopoietic system. The results show that deletion of Atg5 disrupts the transformation of LC3‐I to LC3‐II by lipidation (Figure S2a), a step critical to proceed with the formation of autophagosomes. Deletion of Atg5 significantly reduced the expression level of Sirt3 in the hematopoietic stem and progenitor cells (LSK) and blood precursor cells (Lin^−^) (Figure S2b). Deletion of Atg5 led to increased oxidative stress (Figure S2c) and DNA damage (Figure S2d). Consequently, deletion of Atg5 caused myeloid‐biased differentiation where myeloid differentiation is relatively enhanced over lymphoid differentiation (Figure S2e). These data, together with Atg7 deletion data, support that disruption of autophagy machinery, due to loss of either Atg7 or Atg5, causes loss of Sirt3 and hematopoietic aging.

### 2.5 Autophagy collaborates with Sirt3 to decelerate mouse hematopoietic aging

**FIGURE 4:**
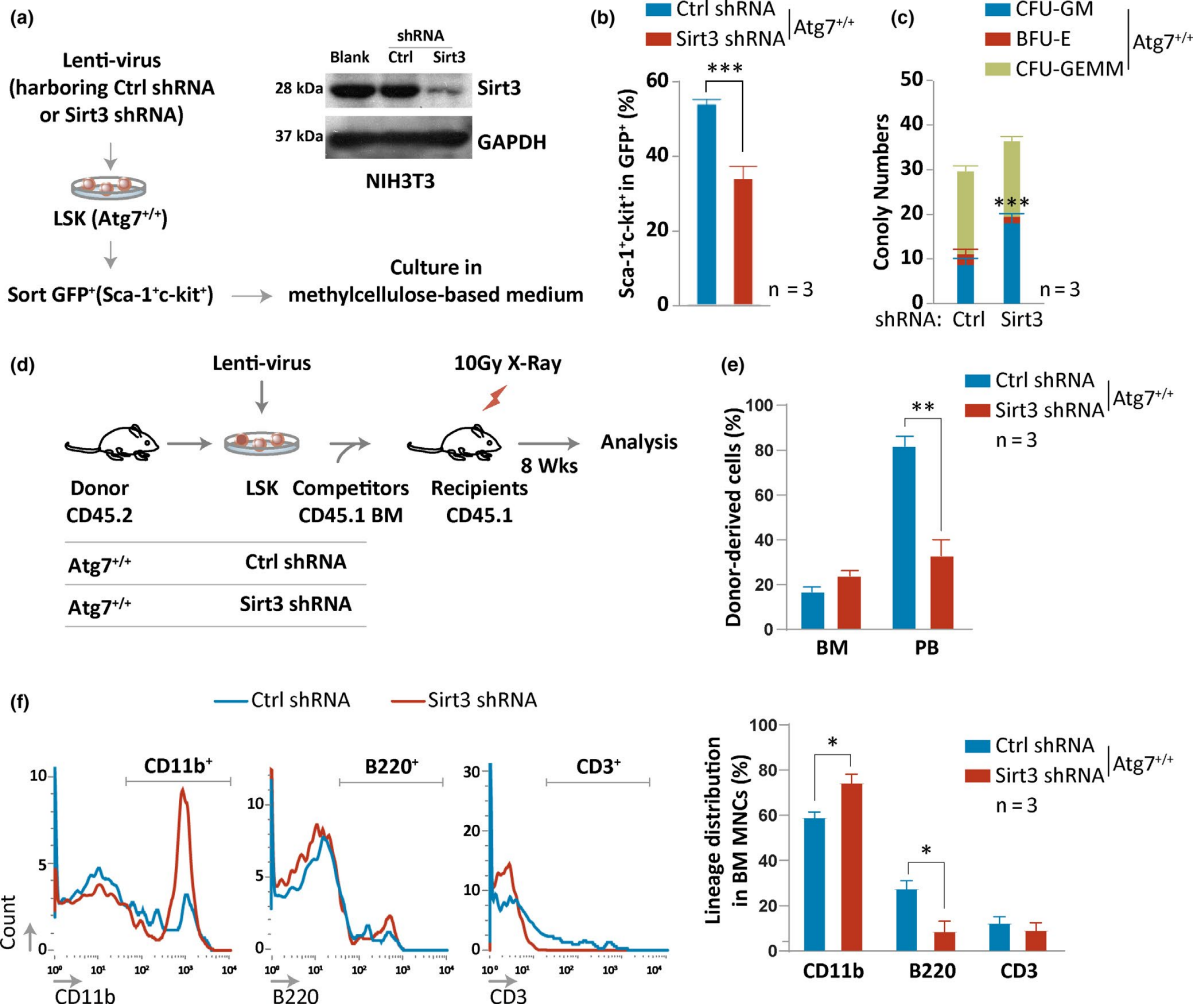
Depletion of Sirt3 accelerates mouse hematopoietic aging. (a‐c) Examination of in vitro self‐renewal capacity of Sirt3‐depleted HSPCs by CFU assay. Sketch showing in vitro knockdown of Sirt3 in HSC‐enriched cells of wild‐type mice by lentivirus infection (a, left); Sirt3 knockdown in the shRNA vector was examined in NIH3T3 cells by Western blotting (a, right); flow cytometric analysis showing reduced percentage of Lin^−^Sca‐1^+^c‐Kit^+^ hematopoietic cells in the Sirt3‐depleted HSC‐enriched cells compared to the control at day 3 after lentivirus infection. GFP^+^ serves a marker in the vector for successful infection of HSPCs (b); CFU assay results with control and Sirt3‐depleted HSC‐enriched cells (c). (d‐f) Examination of donor hematopoietic reconstitution in wild‐type mice transplanted with LSK cells infected with Sirt3 knockdown lentivirus. Sketch showing in vivo knockdown of Sirt3 in the HSC‐enriched cells of wild‐type mice by lentivirus infection (d); flow cytometric examination of donor hematopoietic engraft (e); flow cytometric examination of donor hematopoietic reconstitution in the wild‐type mice (f) with representative flow profiles (left) and statistical results (right)

To examine whether autophagy collaborates with Sirt3 to counteract hematopoietic aging, we began with in vitro colony‐forming assays in which the Sirt3‐depleted HSC‐enriched cells, prepared by infection of the HSC‐enriched cells (GFP^+^) with a lentivirus vector bearing shRNA against the Sirt3 gene, were cultured in methylcellulose medium supplemented with growth factors. Knockdown of Sirt3 with the lentivirus vector, tested by Western blotting for its expression in NIH3T3 cells (Figure 4a), reduced the percentage of HSC‐enriched cells that were sorted against Sca‐1^+^c‐Kit^+^ markers in the GFP^+^ cells of 8‐week‐old mice (Figure 4b). This reduction indicates a reduced proliferation of the Sirt3‐depleted HSC‐enriched cells and hence a significant abnormality in the number of colonies derived from the Sirt3‐depleted HSC‐enriched cells (Figure 4c), suggesting that the in vitro proliferation and self‐renewal capacity of HSC‐enriched cells were reduced through suppression of Sirt3 expression. Next, we transplanted Sirt3‐depleted HSC‐enriched cells that were infected with the lentivirus, together with 10^6^ CD45.1^+^ bone marrow supporting cells, into lethally irradiated mice (Figure 4d). We found that depletion of Sirt3 compromised the donor‐derived hematopoiesis in the wild‐type host mice, with a ~60% reduction in peripheral blood (Figure 4e). Additionally, the flow cytometric data showed a significantly elevated frequency of CD11b myeloid cells and a reduced frequency of B (B220^+^) cells (Figure 4f), which reveals biased myeloid differentiation. From these results, we conclude that the reduction of Sirt3 in HSC‐enriched cells led to an accelerated hematopoietic aging.

**FIGURE 5:**
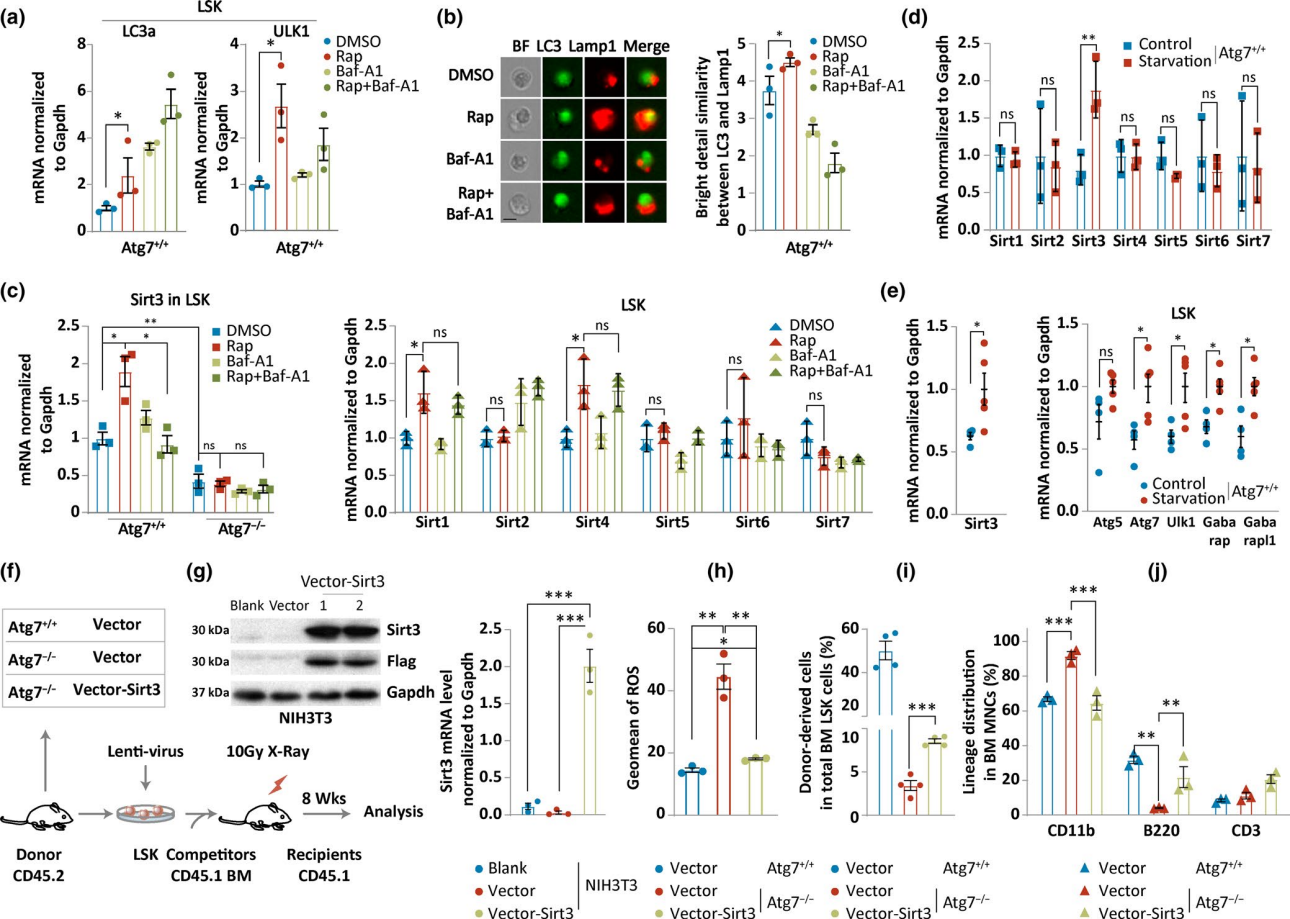
Enhancement of autophagy upregulates Sirt3 expression, and overexpression of Sirt3 reduces oxidative stress and rescues hematopoietic aging in autophagy‐defective mice. (a) Rapamycin upregulates the mammalian autophagy‐essential genes in wild‐type HSC‐enriched hematopoietic cells. Relative expression levels were measured by quantitative PCR. (b) Rapamycin increases the co‐localization between LC3 and Lamp1, an indicator for the formation of autolysosomes, in wild‐type HSC‐enriched hematopoietic cells. The representative images and statistical results were taken and analyzed by Amnis ImageStream image flow cytometer. Rapamycin 200 ng/ml, bafilomycin 10 nM. (c) Quantitative PCR detection of ex vivo autophagy‐induced transcription levels by rapamycin in primary HSC‐enriched hematopoietic cells of wild‐type and Atg7‐deleted mice for Sirt3 (left) and the rest of Sirtuin family members (right). (d) Ex vivo activation of autophagy by starvation selectively upregulates Sirt3 transcription in primary HSC‐enriched hematopoietic cells of wild‐type mice. Transcription levels for Sirtuin family members were detected by quantitative PCR. (e) In vivo activation of autophagy by progressive starvation upregulates Sirt3 transcription. Transcription levels for Sirt3 (left) and autophagy‐essential genes (right) in the HSC‐enriched hematopoietic cells from long‐term progressively starved mice were detected by quantitative PCR. Starvation was achieved by calorie restriction where the amount of feed for the mice was increasingly reduced by 10% of that fed last week, for a total of 4 weeks. (f‐j) Overexpression of Sirt3 reduces oxidative stress and rescues hematopoietic aging in autophagy‐defective mice. Diagrammatic sketch showing the generation of the mouse model with in vivo ectopic overexpression of Sirt3 by lentivirus infection in mouse HSC‐enriched hematopoietic cells, followed by the infected cell transplantation (f); Western blotting analysis of Sirt3 overexpression vector in mammalian cells infected by lentivirus and quantitative result for Sirt3 overexpression in NIH3T3 cells (g); flow cytometric examination of ex vivo ROS levels in the HSC‐enriched cells (h); donor hematopoietic engraft percentage measured 8 weeks after transplantation (i) and hematopoietic reconstitution for myeloid and lymphoid lineages (j) in the Atg7^+/+^ and Atg7^−/−^ host mice infected by control or Sirt3 overexpression lentivirus

To test whether autophagy regulates Sirt3 expression, we triggered an ex vivo autophagy response in wild‐type mouse HSC‐enriched cells with rapamycin, an autophagy inducer that was reported to increase lysosome biogenesis via mTOR signaling (Carroll & Dunlop, 2017). While rapamycin treatment activates autophagy manifested by the upregulation of LC3a and ULK1 at the transcriptional level (Figure 5a), which was further confirmed by enhanced autophagy response measured by Amnis ImageStream imaging flow cytometer for functional co‐localization between autophagosomes (LC3 marker) and lysosomes (Lamp1 marker) (Figure 5b), Sirt3 expression was clearly upregulated in wild‐type HSC‐enriched hematopoietic cells, but was blocked by autophagy inhibitor bafilomycin A1; in contrast, Sirt3 expression was not upregulated in response to autophagy induction in the Atg7‐deleted HSC‐enriched cells (Figure 5c, left). Consistent with RNA sequencing data, most of the Sirtuin family members did not respond to the ex vivo autophagy induction with rapamycin in the wild‐type HSC‐enriched hematopoietic cells, and although Sirt1 and Sirt4 are sensitive to rapamycin, they are not sensitive to the autophagy inhibitor (Figure 5c, right). Furthermore, activation of autophagy by starvation, another frequently used autophagy trigger (Figure S3), selectively upregulated Sirt3 expression, but did not alter the expression levels of the rest of Sirtuin family members in the HSC‐enriched hematopoietic cells (Figure 5d). More importantly, in vivo activation of autophagy by steady starvation with progressive calorie restriction in mice upregulated Sirt3 expression (Figure 5e). The results thus demonstrate that although Sirt3 expression gradually decreases in aging, intervention to enhancement of autophagy is able to fairly selectively upregulate Sirt3 expression in mice.

To confirm whether positive intervention of Sirt3 leads to the reduction in oxidative stress and delay in hematopoietic aging, and to determine whether Sirt3 expression is a downstream event of autophagic protection, we performed ectopic expression of Sirt3 by lentivirus infection in the hematopoietic Atg7‐deleted mice. The HSC‐enriched cells infected with the Sirt3 expression vector, together with 10^6^ CD45.1^+^ bone marrow supporting cells, were transplanted into the lethally irradiated mice (Figure 5f) after the vector was verified by quantitative PCR and Western analysis for effective expression of Sirt3 in the NIH3T3 model cell line (Figure 5g). As a result, ectopic expression of Sirt3 by lentivirus in the Atg7‐deleted mice decreased oxidative stress (Figure 5h) and rescued total bone marrow cellularity (Figure 5i). Remarkably, overexpression of Sirt3 in the Atg7‐deleted mice normalized the generation of myeloid cells and restored the generation of lymphoid cells in the peripheral blood (Figure 5j), effectively reversing the biased myeloid–lymphoid differentiation. Hematopoietic aging caused by a deficit in autophagy was thus rescued by overexpression of Sirt3 in the HSC‐enriched cells of the Atg7‐deleted mice, establishing the proof of principle that enhanced Sirt3 expression reduces oxidative stress and reverses hematopoietic aging due to defects in autophagy.

### 2.6 Activation of autophagy upregulates Sirt3 in human blood

**FIGURE 6:**
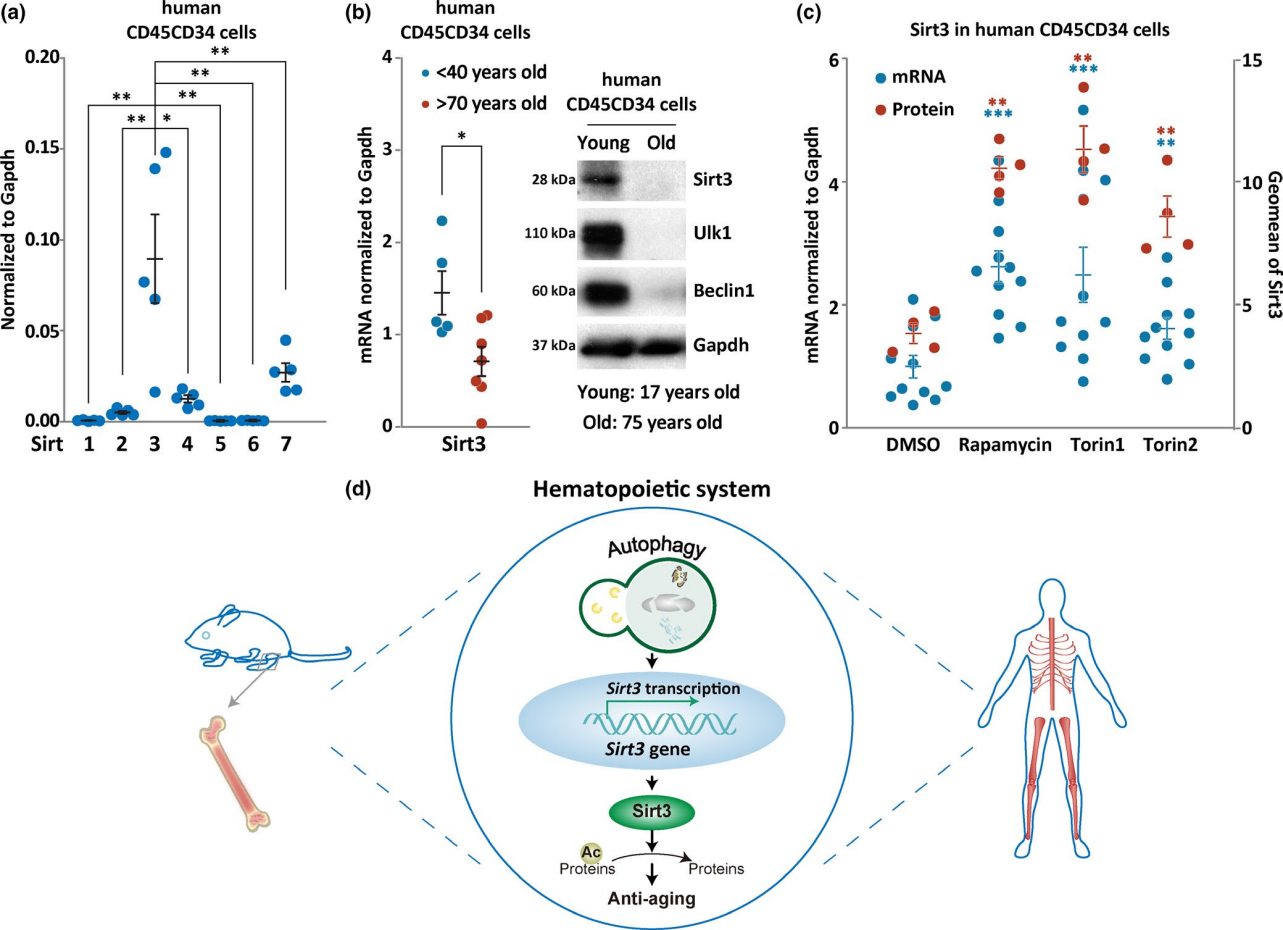
Activation of autophagy by mTOR inhibitors upregulates Sirt3 in human blood. (a) Quantitative RT‐PCR examination of Sirtuin family members in primary CD45CD34 cells from human adults under age of 40. (b) Examination of Sirt3 expression in HSC‐enriched hematopoietic cells from young and old people. Left, quantitative PCR detection of Sirt3 transcription levels in primary CD45CD34 cells from two age human groups. Right, representative images of Western blotting of Sirt3 and key autophagy proteins in primary CD45CD34 cells from young and old individuals. (c) Treatment of the primary CD45CD34 cells from aged human population (40–60 years old) with autophagy inducers upregulates Sirt3 expression. Rapamycin, 200 ng/ml, Torin1, 1 μM; Torin2, 200 nM, treatment for 12 h. Data in blue, mRNA levels; data in red, protein levels. (d) Schematic cartoon illustrating the role of autophagy‐Sirt3 axis in counteracting hematopoietic aging. The dark black arrows represent the anti‐aging axis identified in this study. The light gray arrows represent autophagic pathways counteracting aging in mammalian animals previously reported by other groups

To examine whether the expression pattern of Sirtuin family members in human HSC‐enriched hematopoietic cells is similar to that in mouse HSC‐enriched hematopoietic cells, we measured the transcription levels of the Sirtuin genes in CD45CD34 cells of humans under age of 40 years by quantitative RT‐PCR. The result shows that Sirt3 is the most pronounced member among its family in the HSC‐enriched hematopoietic cells of adult but not aged humans (Figure 6a), which is in consistency with the pattern of wild‐type mice (Figure 3b). Given that hematopoietic autophagy activity is reduced in the aged human population (Figure 1), we intended to test whether Sirt3 also declines in the HSC‐enriched hematopoietic cells of aged human populations. To this end, we measured Sirt3 expression by quantitative RT‐PCR in bone marrow CD45CD34 cells from two different aged human groups (below age of 40 years group and above age of 70 years group). The results show that Sirt3 transcription level was decreased in the group of over 70 years as compared to the group of less 40 years old (Figure 6b, left), a similar pattern to autophagy deterioration in human blood (Figure 1b,c,d). Furthermore, at the translational level, both Sirt3 and two key autophagy proteins, ULK1 and Beclin1, were extremely reduced in the hematopoietic stem cells of the aged individuals (Figure 6b, right), suggesting severe reductions in both autophagy and Sirt3 expression in aged hematopoietic stem cells of humans. To determine whether the expression of Sirt3 is artificially inducible in the aged human blood as it is in the mouse HSC‐enriched cells, aged human CD45CD34 cells were treated with a groups of autophagy inducers that inhibit mTOR, and the results show that induction of autophagy of the HSC‐enriched hematopoietic cells by these inducers all upregulated Sirt3 expression both at transcriptional and translational levels (Figure 6c). The results thus suggest a similar module in human blood in the autophagic regulation of Sirt3 that was identified in mice.

## 3 DISCUSSION

The study presented here provides new insights into autophagic regulation of hematopoietic aging. By examining human blood, we show that although the decrease in peripheral blood counts is mildly progressive, the deterioration of autophagy and reduction of Sirt3 expression are strikingly significant in both hematopoietic cells and their stem and progenitor cells in aged humans. Using two mouse models in which autophagy is genetically impaired in the hematopoietic system, we show that autophagy collaborates with Sirt3, to form a regulatory axis that synergistically delays hematopoietic aging. Furthermore, we demonstrate that the autophagy‐Sirt3 axis is also functional in human hematopoietic system. The summary of this study is illustrated in the cartoon (Figure 6d).

Aging in hematopoietic system not only deteriorates the function of blood cells, but also accelerates aging of non‐hematopoietic organs (Fang et al., 2019; Yuan et al., 2020). Hematopoietic aging in cells, driven by both intrinsic and extrinsic factors, is linked to impaired HSC self‐renewal and reconstitution, and increased hematopoietic malignant incidence. Emerging studies that include increased oxidative stress and compromised DNA damage response (Martin‐Pardillos et al., 2017; Pilzecker et al., 2017), global epigenetic alteration (Akunuru & Geiger, 2016; Florian et al., 2018)) and cell polarity shift (Carrillo‐Garcia & Janzen, 2012; Florian et al., 2018), cellular senescence (Chang et al., 2016), clonal selection of HSCs (Chung & Park, 2017; Pang, Schrier, & Weissman, 2017), frequency of myeloid‐restricted repopulating progenitors (MyRPs) (Yamamoto et al., 2018), as well as chronic inflammation (He et al., 2020), have provided important understanding into the mechanisms of hematopoietic aging.

A recent study on hematopoietic autophagy demonstrated that in aged mice, the majority of HSCs have lower autophagy activity, which contributes to a lower reconstitution potential, with only one‐third of aged HSCs maintaining active autophagy at similar capacity to young mice for self‐renewal and multilineage differentiation (Ho et al., 2017). Here, we show that in aged mice (90 weeks old), the overall capability for HSC reconstitution is significantly low because the peripheral blood counts are markedly reduced and the biased myeloid differentiation is clearly apparent (Figure 2b,c). 10‐week‐old mice with a gene deletion to constitutively suppress autophagy displayed severe hematopoietic aging, mimicking the aging phenotype of 90‐week‐old wild‐type mice (Figure 2b). However, in contrast to these observations in mice, peripheral blood counts only display a mildly progressive decline in aging humans covering a broad age range from 20 to 90 years old (Figure 1a). Nevertheless, autophagy‐essential genes and their function in human autophagy machinery are strongly inhibited in aged individuals (Figure 1b,c,d; Figure 6b) to a degree comparable with that of the autophagy‐defective mouse model (Cao, Cai, et al., 2015; Cao, Zhang, et al., 2015). These results suggest that functional deterioration of the hematopoietic system may largely be attributed to insufficient autophagy activity in humans.

Sirt3 increases the activity of antioxidants, such as superoxide dismutase 2 (SOD2), and promotes ROS scavenging (Qiu, Brown, Hirschey, Verdin, & Chen, 2010; Someya et al., 2010; Tao et al., 2010; Tseng, Shieh, & Wang, 2013; Yu, Dittenhafer‐Reed, & Denu, 2012). Decreased expression of Sirt3 in aged HSCs is associated with a concomitant repression of mitochondrial protective programs, which can result in impaired function of the Sirt3‐directed mitochondrial unfolded protein response pathway (Brown et al., 2013). Sirt3 is indispensable under stress or in aged mice, and it is downregulated with age, contributing to increased ROS levels in aged HSCs (Brown et al., 2013). In this study, we find that Sirt3 is the major Sirtuin family member in the HSC‐enriched hematopoietic cells of both mice (Figure 3) and humans (Figure 6a). Transcription of Sirt3 relies on autophagy and is progressively reduced from early to late stages in the hematopoietic hierarchy (Figure 3e), showing an accelerated drop in autophagy‐defective mice, with expression at 10 weeks of age comparable to that of the wild‐type mice at 84 weeks of age (Figure 3h,i). An ex vivo study showed that Sirt3 is the only member in Sirtuin family that effectively responds to multiple autophagy inducers (Figure 5c,d). Similarly, an in vivo study with long‐term calorie restriction further revealed that restriction of food intake upregulates Sirt3, along with the upregulation of autophagy (Figure 5e). These results indicate that Sirt3 expression relies heavily on intact autophagy machinery, and Sirt3 decline is associated with low autophagy capacity, whereas activation of autophagy reverses Sirt3 decline. We thus conclude that aging‐related suppression of Sirt3 is attributed to the deterioration of the capacity for autophagy. We previous found that in a non‐aging blood cancer cell line, Sirt3 appears to be associated with elevated oxidative stress and is downregulated by autophagy (Fang et al., 2016). The discrepancy in the role and autophagic regulation of Sirt3 between normal and malignant hematopoietic cells is unknown and remains to be explored in the future.

Our bioinformatic analysis and experimental validation identified Sirt3 as a downstream effector of autophagy for regulation of hematopoietic aging. Functional analysis demonstrated that induction of autophagy upregulates Sirt3 expression; Sirt3 overexpression reduces oxidative stress and reverses hematopoietic aging in autophagy‐defective HSC‐enriched hematopoietic cells. These data together suggest that autophagy, as the upstream regulatory machinery, activates Sirt3 expression forming an autophagy‐Sirt3 axis in hematopoietic cells for coordinated control of oxidative stress and hematopoietic aging.

Sirt3 is enriched in mitochondria in the cell and initiates metabolic adaptations to enhance mitochondrial management of oxidative stress inducers; therefore, Sirt3 positively regulates mitophagy (Meng et al., 2019). Mitophagy is regulated by multiple mechanisms including post‐translational modification of a long array of enzymes that catalyze phosphorylation, acetylation, and deacetylation, etc. (Wang, Qi, Tang, & Shen, 2020). Increased mitochondria are often a cause of low capacity of mitophagy that promotes aging (Bakula & Scheibye‐Knudsen, 2020). Our present study with Atg7‐deleted mice indicates an increase of mitochondrial mass (Figure 2d) and a decrease in Sirt3 (Figure 3), but activation of autophagy did not reduce Sirt3 levels and Sirt3 prevented premature hematopoietic aging in an autophagy‐disrupted mouse model, and we conclude that the evidence is compelling to support the hypothesis that the autophagy‐Sirt3 axis delays hematopoietic aging independently of the mitophagy pathway.

Sirt3 regulates aging via its deacetylation capacity (Brown et al., 2013; Shimazu et al., 2010; Someya et al., 2010). Although overwhelming studies indicate Sirt3 functions in the mitochondria due to its mitochondrial localization, a previous study shows that in humans, both the full‐length and processed forms of Sirt3 target H4‐K16 for deacetylation in vitro and can deacetylate H4‐K16 in vivo when recruited to a gene, and Sirt3 is transported from the nucleus to the mitochondria upon cellular stress (Scher, Vaquero, & Reinberg, 2007). A recent work with systematic study suggests that human Sirt3 displays class‐selective histone de‐β‐hydroxybutyrylase activities with preference for H3 K4, K9, K18, K23, K27, and H4 K16, but not for H4 K5, K8, and K12, which distinguishes it from HDACs, another group of deacetylation enzymes (Zhang et al., 2019). These reports suggest that Sirt3 is able to deacetylate proteins in the nucleus, not just in the mitochondria, albeit a transient nuclear presence for full‐length Sirt3.

To answer whether Sirt3 deacetylation activity on nuclear proteins depends on autophagy machinery, we measured histone acetylation levels in the bone marrow mononuclear cells of the wild‐type and Atg7‐deleted mice, and the results show that histone acetylation was highly accumulated in the Atg7‐deleted mice (Figure S4). Furthermore, transcriptional profiling of hematopoietic stem and progenitor cells indicates that Atg7 deletion does not alter the expression levels of all eleven members of HDAC family (Figure S5). These data appear to be in agreement with previous reports on nuclear autophagy that targets nuclear components for degradation (Luo, Zhao, Song, Cheng, & Zhou, 2016; Papandreou & Tavernarakis, 2019). Our results thus propose that Sirt3 more likely contributes to the deacetylation of histone proteins in hematopoietic cells in the context of autophagic regulation, and the acetylation activity of Sirt3 appears to be autophagy‐dependent in hematopoietic aging.

Similar to our observations in mice, Sirt3 is also highly expressed in the HSC‐enriched hematopoietic cells of young humans, but it is highly suppressed in the hematopoietic system of aged humans (Figure 6b). In accordance with the observation that in vivo activation of autophagy by caloric restriction upregulates Sirt3 expression in mice (Figure 5e), the induction of autophagy by various mTOR inhibitors effectively upregulates Sirt3 in the HSC‐enriched hematopoietic cells of humans (Figure 6c). This suggests that the autophagy‐Sirt3 axis regulating aging is similarly present in the mouse and human hematopoietic system (Figure 6d). Therefore, our finding provides promising translational avenues for decelerating human hematopoietic aging by enhancing the autophagy‐Sirt3 axis with autophagy inducers, or by enhancing Sirt3 expression.

Although our study has established the functional connection between autophagy and Sirt3 transcription, there are missing parts in the regulatory autophagy‐Sirt3 axis. In particular, the direct target of autophagy, which may negatively regulates Sirt3 expression in the nucleus, has yet to be identified. Our future work will continue the search for the direct targets of autophagy in the anti‐aging regulatory axis.

## 4 EXPERIMENTAL PROCEDURES

### 4.1 Clinical records and human samples

The clinical records in human blood test were provided by the Department of Orthopaedics, the Second Affiliated Hospital of Soochow University. Human blood samples for autophagic analysis were obtained from volunteer donors with their consent. All experiments with human samples and clinical records were approved by the Research Management Office of the Affiliated Hospital of Soochow University. Human mononuclear cells were enriched by equilibrium centrifugation over a cushion of Ficoll‐Paque Plus (17544652, GE Healthcare) by density gradient centrifugation. Human HSPCs were purified from bone marrow CD45‐positive cells by human CD34‐positive selection kit (18056, STEMCELL Technologies). Human HSPCs were cultured in IMDM (SH30228, GE Healthcare) with 10% FBS, 1% penicillin/streptomycin, SCF (100 ng/ml), Flt3L (100 ng/ml), IL6 (20 ng/ml), IL3 (20 ng/ml), and G‐CSF (20 ng/ml) (PeproTech).

### 4.2 Mice

The generation of Atg7^flox/flox^ mice has been previously described (Cao, Zhang, et al., 2015; Komatsu et al., 2005). Atg5^flox^ mice were provided by Dr. Noboru Mizushima, Japan (Hara et al., 2006). Vav‐iCre transgenic mice were purchased from the Jackson Laboratory. All mice were bred on a C57BL/6 genetic background. Whole blood was collected from the mouse orbit after anesthesia, subjected to an automated blood count (ADVIA 2120i). For calorie restriction, mice were starved by progressive reducing 10% food intake per week for 1 month. All animal experiments were reviewed and approved by the Institutional Committee on Animal Welfare Protection and Ethics of Soochow University.

### 4.3 Chemicals and biological reagents

Primary hematopoietic cells from mice and humans were treated with rapamycin (S1039, Selleckchem), bafilomycin A1 (196000, Sigma), Torin1 (orb146133, Biorbyt), and Torin2 (orb146132, Biorbyt) between 12 and 16 h. LSK cells were starved by HBSS (sh30015, HyClone) ex vivo by 2 h. The information of biological reagents is provided in Table S1.

### 4.4 Flow cytometry, cell sorting, and colony analysis

Single‐cell suspensions from BM and PB were stained with panels of fluorochrome‐conjugated antibodies (Table S1). Flow cytometric analysis of HSPCs was performed as previously described (Cao, Cai, et al., 2015). The analyses were performed using a FACSCalibur or Beckman Coulter Gallios. All data were analyzed using FlowJo software or Kaluza. BM lin^−^ cells were sorted using Mouse Lineage Cell Depletion Kit (130090858, Miltenyi Biotec). Cells were sorted by BD FACSAria III. LSK cells were plated in triplicate in methylcellulose medium (03134, STEMCELL Technologies) supplemented with mouse stem cell factor (mSCF) (100 ng/ml), mouse IL‐3 (mIL‐3) (10 ng/ml), mouse erythropoietin (mEPO) (4 U/ml), mouse thrombopoietin (mTPO) (50 ng/ml), mouse granulocyte–macrophage CSF (mGM‐CSF) (10 ng/ml), and human IL‐6 (50 ng/ml, PeproTech). The cells were then scored on day 10 of the cultures at 37°C and 5% CO_2_.

### 4.5 Cell proliferation

BrdU (19160, Merck Millipore) was injected daily into the peritoneum (100 mg/kg body weight in 0.9% NaCl) for 7 days, with the last injection 2 h prior to harvesting BM. BrdU‐positive nuclei were detected with BD Pharmingen™ BrdU Flow Kits (559619, BD Pharmingen), as its manual describes. Ki‐67 (652417, BioLegend)‐labeled cells and Hoechst (H3570, Thermo Fisher Scientific)‐stained nuclei were counted in LSK cells by flow cytometry.

### 4.6 Lentiviral transduction of HSC‐enriched cells

As previously described (Cao, Cai, et al., 2015), sorted LSK cells were prestimulated for 5–10 h in a 96‐well dish (167008, Thermo Fisher Scientific) supplemented with 10% FBS (10099‐141, Gibco), 1% penicillin/streptomycin (AP231, AS325, Beijing Dingguo Changsheng Biotechnology), IL3 (20 ng/ml), IL6(20 ng/ml), TPO (50 ng/ml), Flt3L (50 ng/ml), and SCF (100 ng/ml, PeproTech). Sirt3 gene was cloned into the pCAG lentiviral construct. Sirt3 shRNA (GGAAGACATATGGGCTGATGT) was purchased from GenePharma. The lentiviral media were added to LSK cells in a 96‐well plate which is coated by fibronectin (07159, STEMCELL Technologies). NIH3T3 and 293T cell lines were purchased from ATCC. They were cultured in 10% FBS DMEM (10‐013‐CVR, Corning).

### 4.7 Transplantation assays

LSK single‐cell suspensions were harvested from 6‐week‐old Atg7^+/+^ and Atg7^−/−^ mice, and to be infected by lentivirus. 1 × 10^3^ GFP^+^ cells and 1 × 10^5^ CD45.1 BM competitor cells were injected into lethally irradiated (10 Gy) WT recipients (CD45.1) through the tail vein. The recipient mice were sacrificed at 8 weeks after transplantation.

### 4.8 Immunofluorescence

Cells were fixed in 4% paraformaldehyde (8009628, Sinopharm Chemical Reagent) or 15 min, permeabilized in 0.5% Triton X‐100 (DH351‐2, Beijing Dingguo Changsheng Biotechnology) in PBS for 5 min, blocked with 4% goat serum in PBS for 60 min, and incubated with rabbit monoclonal anti‐Sirt3 (Ab86671, Abcam) overnight at 4°C. DyLight 488‐conjugated goat anti‐rabbit (GAR4882, Multi Sciences) was used as a secondary antibody. Samples were stained with DAPI (C1002, Beyotime) before photographed on a fluorescence microscope (FV1000MPE‐share).

### 4.9 RNA‐seq analysis

LSK cells were sorted from 10‐week‐old Atg7^+/+^ and 10‐week‐old Atg7^−/−^ mice. Sequencing libraries were generated using NEBNext® UltraTM RNA Library Prep Kit for Illumina® (NEB, USA) following the manufacturer's recommendations and followed by sequencing with on an Illumina HiSeq platform and 125 bp/150 bp paired‐end reads were generated. Approximately 40 million paired‐end reads per sample were aligned to the mouse genome (GRCm38/mm10). HTSeq v0.6.0 was used to count the reads numbers mapped to each gene. FPKM of each gene was calculated based on the length of the gene and reads count mapped to this gene. Differential expression analysis of two conditions/groups (two biological replicates per condition) was performed using the DESeq2 R package (1.10.1). DESeq2 provides statistical routines for determining differential expression in digital gene expression data using a model based on the negative binomial distribution. The resulting *p*‐values were adjusted using the Benjamini and Hochberg's approach for controlling the false discovery rate. Genes with an adjusted *p*‐value <0.05 and fold change >2 found by DESeq2 were assigned as differentially expressed. LSK cells were sorted from 10‐month‐old Atg7^+/+^ and Atg7^−/−^ mice. Sequencing libraries were generated using NEBNext® UltraTM RNA Library Prep Kit for Illumina® (NEB, USA) following the manufacturer's recommendations, and the quantity and quality of them were assessed by Qubit 2.0 Fluorometer and Agilent 2100 bioanalyzer and followed by sequencing with on an Illumina HiSeq PE150 platform and 150 bp paired‐end reads were generated. Approximately 40 million paired‐end reads per sample were aligned to the mouse genome (GRCm38/mm10). HTSeq v0.6.0 (Anders, Pyl, & Huber, 2015) was used to count the reads numbers mapped to each gene. FPKM of each gene was calculated based on the length of the gene and reads count mapped to this gene. The expression levels of all genes were calculated by RSEM v1.2.28 (Li & Dewey, 2011). Differential expression analysis of two conditions/groups (two biological replicates per condition) was performed using the edgeR package (v3.12.1) (Robinson, McCarthy, & Smyth, 2010).The edgeR provides statistical routines for determining differential expression in digital gene expression data using a model based on the negative binomial distribution. The resulting *p*‐values were adjusted using the Benjamini and Hochberg's approach for controlling the false discovery rate (Benjamini & Yekutieli, 2005). Genes with an *p*‐value <0.05 and fold change >2 found by edgeR were assigned as differentially expressed and visualized as volcano map. In addition, the differentially expressed pattern of Sirtuin gene family was analyzed by using R language (v3.4.3).

### 4.10 qRT‐PCR analysis

Total RNA was extracted with MicroElute Total RNA Kit (R6831, Omega), and cDNA was synthesized using the RevertAid cDNA Synthesis Kit (K1622, Thermo Fisher Scientific) according to the manufacturer's instructions. RT‐qPCR was performed in triplicate using Roche LightCycler 480 Instrument II with the LightCycler 480 SYBR Green I Master (04707516001, Roche). Expression of the gene of interest was normalized to the housekeeping gene GAPDH using the 2^–ΔΔCt^ method. All RT‐qPCR primers used are listed in Table S2.

### 4.11 Western blot analysis

Cells were harvested from 10‐week‐old mice. Lysates from cells were prepared using cell lysis (9803S, Cell Signaling Technology) for Western blotting as previously described (Cao, Cai, et al., 2015). Antibodies used are ATG7 (ab133528, Abcam), ATG5 (ET1611‐38, HuaBio), LC3 (nb100‐2220, Novus), GAPDH (60004‐1, Proteintech), SIRT3 (5490, Cell Signaling Technology), and secondary antibodies (7074S, 7076S, Cell Signaling Technology).

### 4.12 ImageStream analysis

Image flow cytometric analysis of co‐localization of CD34 (555821, BD Pharmingen), CD45 (563879, BD Pharmingen), LC3 (14079S, Cell Signaling Technology), and Lamp1 (ab24170, Abcam) was performed with Amnis ImageStream Image Flow Cytometer (Amnis, Merck Millipore). Cells were first stained with CD34 and CD45 for 30 min. After fixation and permeabilization using 4% PFA and 0.1% saponin (S7900, Sigma), cells were stained with primary antibody against LC3 and Lamp1 for 30 min and then stained with DyLight 488 conjugated of goat anti‐rabbit IgG(H+L) or DyLight 649 conjugated of goat anti‐mouse IgG(H+L) (GAR6492, Multi Sciences) for 30 min; then, cells were explored to ImageStreamX Mark II machine for image flow cytometry. Samples were visualized and analyzed for the expression of marker proteins with IDEAS 6.0 software (Amnis, Merck Millipore). Scale bar: 10 μM.

### 4.13 Statistical analysis

The statistical significance of differences was calculated for Pearson correlation coefficient of clinical data using SPSS version 23.0 or Student's unpaired *t* test for experiments with animals and all primary samples including animals and humans. Graphs containing error bars show the mean ±SEM. Statistical significance is represented as *p < 0.05, **p < 0.01, ***p < 0.001 and not significant (ns).

## CONFLICTS OF INTEREST

The authors have declared no conflicts of interest.

## AUTHOR CONTRIBUTIONS

YF and JW conceived the project. YF designed the study. YF, NA, LZ, YG, JQ, GJ, and RZ performed the experiments. GZ, YF, and XY analyzed the RNA sequencing data. WW and LX performed mouse genotyping. YF, NY, SP, YZ, and JW discussed and analyzed the data. YF and JW wrote the manuscript. All authors read and approved the manuscript.

## Supporting information

Supplementary Material

## Data Availability

The authors declare that the main data supporting the findings of this study are available within the article and these supplementary information files. The RNA sequencing data have been deposited in GenBank database with an accession number PRJNA506815 (http://www.ncbi.nlm.nih.gov/biopr​oject/​506815).
