# Supplementary material for: Autophagy‐Sirt3 axis decelerates hematopoietic aging
Source: Aging Cell. 2020 Sep 20;19(10):e13232. doi: 10.1111/acel.13232 (PMC7576273; doi:10.1111/acel.13232)
Supplement: Supplementary file 1 — Supplementary Material [file ACEL-19-e13232-s001.pdf]

# Autophagy-Sirt3 axis decelerates hematopoietic aging

## Supplementary Data

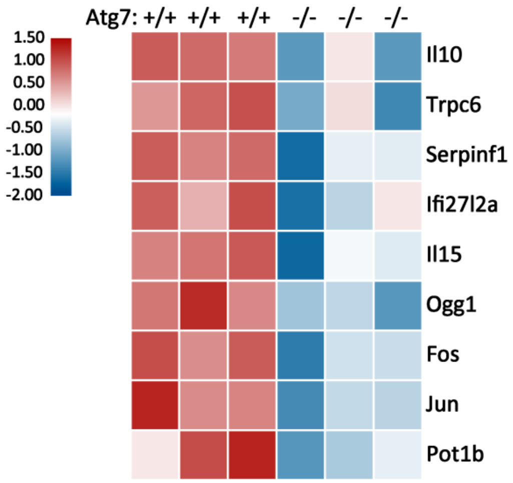


**Supplementary Figure S1. Heatmap of anti-aging genes between Atg7$^{+/+}$ and Atg7$^{-/-}$ HSC-enriched cells.** The result shows that deletion autophagy-essential gene Atg7 leads to downregulation of anti-aging markers in the HSC-enriched hematopoietic cells of 10-week-mice.

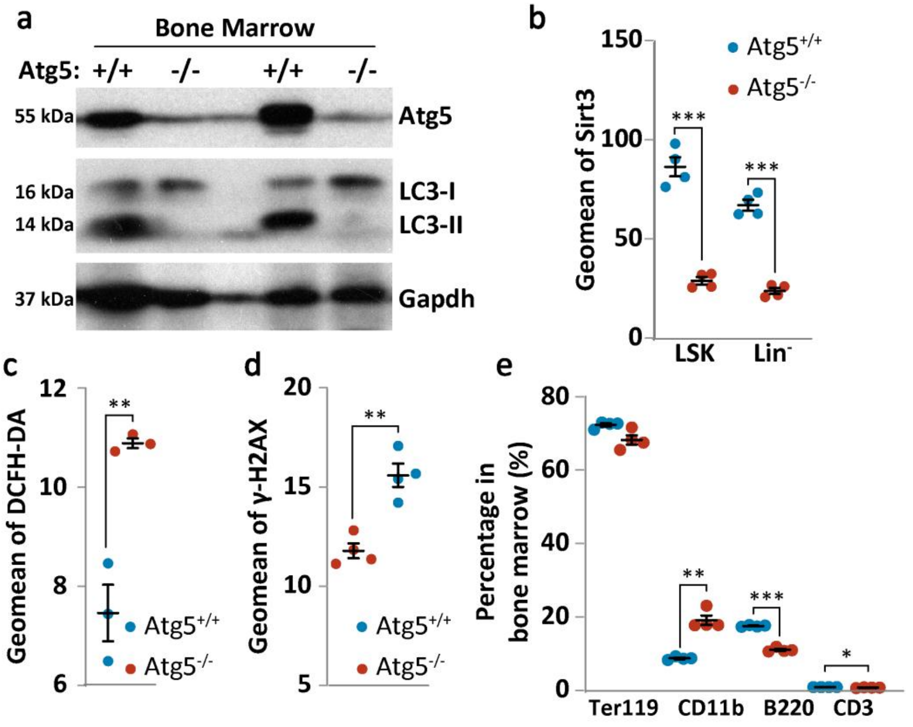


**Supplementary Figure S2**. **Deletion of Atg5 in hematopoietic system reduces Sirt3 expression and causes faster aging**. **a**. Identification of the atg5 deletion and autophagy disruption in mouse hematopoietic system by Western blotting. Atg5$^{flox}$ mice was used to cross Vav-iCre to generate Atg5-deleted mice in the hematopoietic system. Western blot indicates the disruption of Atg5 gene and impaired lipidation of LC3-I to LC3-II in the bone marrow mononuclear cells. **b**. Flow cytometric measurement of Sirt3 protein levels in the hematopoietic stem and progenitor cells of the wild-type and Agt5-deleted mice. **c**. Flow cytometric analysis of DCFH-DA levels in the hematopoietic stem and progenitor cells of wild-type and Atg5-deleted mice. **d**. Flow cytometric analysis of γ- H2AX levels in the wild-type and Atg5-deleted mice. **e**. Flow cytometric analysis of myeloid-biased hematopoietic differentiation in wild-type and Atg5-deleted mice. Mouse numbers in each group are shown with the number of dots. *$P$<0.05, **$P$<0.005, ***$P$<0.001.

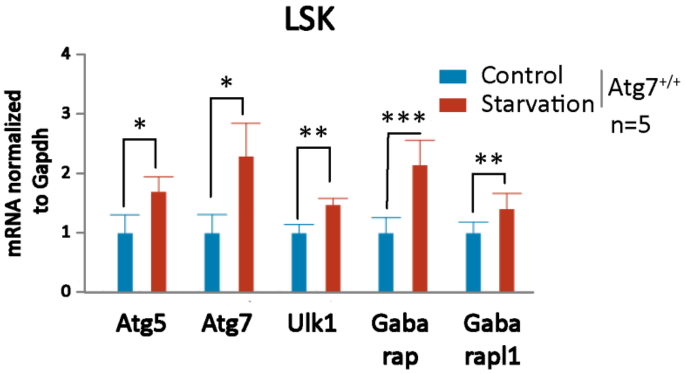


**Supplementary Figure S3.** ***Ex vivo*** **activation of autophagy by starvation in primary HSC-enriched hematopoietic cells of wild-type mice.** Transcription levels for autophagy-essential genes in bone marrow LSK population were detected by quantitative PCR. Primer information is listed in the Supplementary Table 2. *$P$<0.05, **$P$<0.005, ***$P$<0.001.

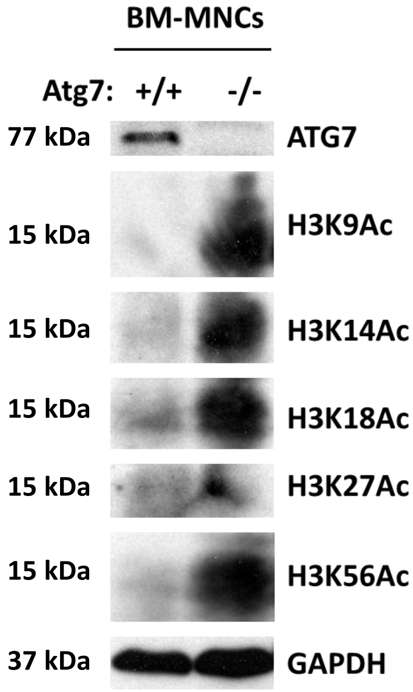


**Supplementary Figure S4. Deletion of Atg7 accumulates acetylation levels of Histone 3 protein.** Western blotting results indicate that in the bone marrow mononuclear cells of the Atg7-deleted mice, acetylation at lysine 9, 14, 18, 27 and 56 was increased.

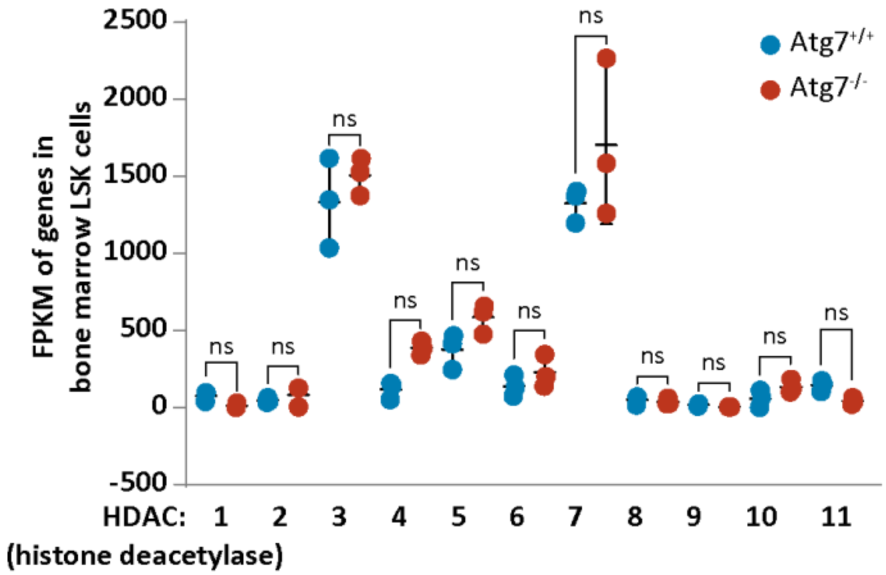


**Supplementary Figure S5. Deletion of Atg7 does not change expression levels of all eleven members of HDAC family in the hematopoietic stem and progenitor cells of the mouse.** FPKM values of HADCs including eleven members of $Atg7^{+/+}$ and $Atg7^{-/-}$ LSK cells. FPKM: fragments per kilobase of exon model per million reads mapped

**Supplementary Table S1 Key reagents information**

| Reagents name | Company | Catalog No. |
|---|---|---|
| CD45.1-PerCP-Cy5.5 | eBioscience | 45-0453-82 |
| CD45.2-BV421 | BD pharmingen | 562895 |
| CD3-APC-Cy7 | BD pharmingen | 557596 |
| B220-PE-Cy7 | BD Pharmingen | 552772 |
| TER119-PE | eBioscience | 12-5921-81 |
| Scal-1-APC | eBioscience | 17-5981-82 |
| CD117(c-kit)-APC-eFluor 780 | eBioscience | 47-1172-82 |
| ROS(DCFH-DA) | Sigma | D6883 |
| Mitotracker deep red | Invitrogen | M22426 |
| y-H2AX-Alexa Fluor® 488 | Cell Signalling Technology | 20304S |
| Ubiquitin | Abcam | ab115637 |
| CellROX™ Deep Red Flow Cytometry Assay Kit | Thermo | C10491 |

**Supplementary Table S2 Primers for quantitative PCR detection of gene expression**

| **Mouse** | Primer sequence |
|---|---|
| mus Tert-F | GCACTTTGGTTGCCCAATG |
| mus Tert-R | GCACGTTTCTCTCGTTGCG |
| mus Terf1-F | TGCGGGCTGGATGCTC |
| mus Terf1-R | TCAGGGCTGATTCCAAGGGTG |
| mus Terf2-F | GAGGCGGGAGCAGCGATAG |
| mus Terf2-R | CCCAAGGGCCTGACAAGCAAC |
| GAPDH-F | AGCTTGTCATCAACGGGAAG |
| GAPDH-R | TTTGATGTTAGTGGGGTCTCG |
| mSirt1-F | GTAATGTGAGGAGTCAGCAC |
| mSirt1-R | TTGGACATTACCACGTCTGC |
| mSirt2-F | GCCTGGGTTCCCAAAAGGAG |
| mSirt2-R | GAGCGGAAGTCAGGGATACC |
| mSirt3-F | CATTCGGGCTGACGTGATG |
| mSirt3-R | AACCACATGCAGCAAGAACCT |
| mSirt4-F | GTGGAAGAATAAGAATGAGCGGA |
| mSirt4-R | GGCACAAATAACCCCGAGG |
| mSirt5-F | CTCCGGGCCGATTCATTTCC |
| mSirt5-R | GCGTTCGCAAAACACTTCCG |
| mSirt6-F | ATGTCGGTGAATTATGCAGCA |
| mSirt6-R | GCTGGAGGACTGCCACATTA |
| mSirt7-F | AGCATCACCCGTTTGCATGA |
| mSirt7-R | GGCAGTACGCTCAGTCACAT |
| mATG7-F | GTTCGCCCCCTTTAATAGTGC |
| mATG7-R | TGAACTCCAACGTCAAGCGG |
| LC3A-F | GACCGCTGTAAGGAGGTGC |
| LC3A-R | CTTGACCAACTCGCTCATGTTA |
| LC3B-F | TTATAGAGCGATACAAGGGGGAG |
| LC3B-R | CGCCGTCTGATTATCTTGATGAG |
| ATG5-F | ATTCCAACGTGCTTTACTCTCTATC |
| ATG5-R | AAACCAAATCTCACTAACATCTTCT |
| mULK1-F | TGGAGGTGGCCGTCAAATG |
| mULK1-R | CGCATAGTGTGCAGGTAGTC |
| mGabarap-F | AAGAGGAGCATCCGTTCGAGA |
| mGabarap-R | GCTTTGGGGGCTTTTTCCAC |
| mGabarapl1-F | GGACCACCCCTTCGAGTATC |
| mGabarapl1-R | CCTCTTATCCAGATCAGGGACC |

| **Human** | Primer sequence |
|---|---|
| hGAPDH-F | CGACCACTTTGTCAAGCTCA |
| hGAPDH-R | GGGTCTTACTCCTTGGAGGC |
| hULK1-F | TCGAGTTCTCCCGCAAGG |
| hULK1-R | CGTCTGAGACTTGGCGAGGT |
| hAtg5-F | CAACTTGTTTCACGCTATATCAGG |
| hAtg5-R | CACTTTGTCAGTTACCAA CGTCA |
| hAtg7-F | TAATGTCCTTCCCGTCAGCCT |
| hAtg7-R | TCATGTCCCAGATCTCAGCAG |
| hAtg4b-F | GTCCTGAACCTGTCCCTAGATTC |
| hAtg4b-R | CCCCGACCCCAGGATTTTC |
| hAtg12-F | AAGTGGGCAGTAGAGCGAAC |
| hAtg12-R | CCATCACTGCCAAAACACTCA |
| hLC3b-F | TGTCCGACTTATTCGAGAGCAGCA |
| hLC3b-R | TTCACCAACAGGAAGAAGGCCTGA |
| hP62-F | GACTACGACTTGTGTAGCGTC |
| hP62-R | AGTGTCCGTGTTTCACCTTCC |
| hFUNDC1-F | CCTCCCCAAGACTATGAAAGTGA |
| hFUNDC1-R | AAACACTCGATTCCACCACTG |
| hLamp1-F | TCTCAGTGAACTACGACACCA |
| hLamp1-R | AGTGTATGTCCTCTTCCAAAAGC |
| hLamp2a-F | GCACAGTGAGCACAAATGAGT |
| hLamp2a-R | CAGTGGTGTGTATGGTGGGT |
| hGabarap-F | AGAAGAGCATCCGTTCGAGAA |
| hGabarap-R | CCAGGTCTCCTATCCGAGCTT |
| hGabarapl1-F | ATGAAGTTCCAGTACAAGGAGGA |
| hGabarapl1-R | GCTTTTGGAGCCTTCTCTACAAT |
| mSirt1-F | GTAATGTGAGGAGTCAGCAC |
| mSirt1-R | TTGGACATTACCACGTCTGC |
| mSirt2-F | GCCTGGGTTCCCAAAAGGAG |
| mSirt2-R | GAGCGGAAGTCAGGGATACC |
| hSirt3-F | CATTCGGGCTGACGTGATG |
| hSirt3-R | AACCACATGCAGCAAGAACCT |
| mSirt4-F | GTGGAAGAATAAGAATGAGCGGA |
| mSirt4-R | GGCACAAATAACCCCGAGG |
| mSirt5-F | CTCCGGGCCGATTCATTTCC |
| mSirt5-R | GCGTTCGCAAAACACTTCCG |
| mSirt6-F | ATGTCGGTGAATTATGCAGCA |
| mSirt6-R | GCTGGAGGACTGCCACATTA |
| mSirt7-F | AGCATCACCCGTTTGCATGA |
| mSirt7-R | GGCAGTACGCTCAGTCACAT |
| hBecn1-F | TGTCCACAGAAAGTGCCAACA |
| hBecn1-R | CCTCACAGAGTGGGTGATCCA |
